# Estimating differential expression from multiple indicators

**DOI:** 10.1093/nar/gku158

**Published:** 2014-02-27

**Authors:** Sten Ilmjärv, Christian Ansgar Hundahl, Riin Reimets, Margus Niitsoo, Raivo Kolde, Jaak Vilo, Eero Vasar, Hendrik Luuk

**Affiliations:** ^1^Department of Physiology, Institute of Biomedicine and Translational Medicine, University of Tartu, Tartu, Estonia, ^2^Quretec Ltd, Tartu, Estonia, ^3^Centre for Excellence in Translational Medicine, University of Tartu, Tartu, Estonia, ^4^Department of Neuroscience and Pharmacology, Faculty of Health Sciences, University of Copenhagen, Copenhagen, Denmark and ^5^Department of Computer Science, University of Tartu, Tartu, Estonia

## Abstract

Regardless of the advent of high-throughput sequencing, microarrays remain central in current biomedical research. Conventional microarray analysis pipelines apply data reduction before the estimation of differential expression, which is likely to render the estimates susceptible to noise from signal summarization and reduce statistical power. We present a probe-level framework, which capitalizes on the high number of concurrent measurements to provide more robust differential expression estimates. The framework naturally extends to various experimental designs and target categories (e.g. transcripts, genes, genomic regions) as well as small sample sizes. Benchmarking in relation to popular microarray and RNA-sequencing data-analysis pipelines indicated high and stable performance on the Microarray Quality Control dataset and in a cell-culture model of hypoxia. Experimental-data-exhibiting long-range epigenetic silencing of gene expression was used to demonstrate the efficacy of detecting differential expression of genomic regions, a level of analysis not embraced by conventional workflows. Finally, we designed and conducted an experiment to identify hypothermia-responsive genes in terms of monotonic time-response. As a novel insight, hypothermia-dependent up-regulation of multiple genes of two major antioxidant pathways was identified and verified by quantitative real-time PCR.

## INTRODUCTION

Regardless of the advent of high-throughput sequencing, microarrays remain central in current biomedical research, as their maturity arguably enables more accurate transcriptional profiling in some situations (1,2) and facilitated analysis due to lower data volume. As microarrays continue to be a cost-effective tool for probing large-scale gene expression, they are likely to remain the method of choice in focused clinical and diagnostic settings not seeking to identify novel sequence variants. Moreover, public databases such as ArrayExpress and Gene Expression Omnibus (3,4) contain microarray data from tens of thousands of experiments and this vast data source will remain uncontested by sequencing datasets in the coming years. State-of-the-art high-density microarrays such as Affymetrix Gene® and Exon® arrays produce millions of probe-level signals representing most of the transcriptome. Although it is desirable to make optimal use of this rich information, the appearance of modern high-density microarrays has not led to the establishment of dedicated analysis methodologies. On one hand, thousands of genes are interrogated simultaneously making it possible to ‘borrow’ information across genes when estimating differential expression (DE) (5). On the other hand, as each gene is interrogated dozens of times replicated measurements can be integrated to yield a more robust DE estimate. As a rule, however, probe-level information is summarized into probe-set values precluding its use directly in computing DE estimates (6). Such practice is not optimal as the number of variables available for inferring DE is reduced, which, in turn, can lead to the reduction in statistical power. This issue can become critical when the number of replicates per treatment is small as in most microarray experiments.

Here, we introduce a novel DE analysis methodology designed to take advantage of the high number of concurrent measurements provided by high-density microarrays. The estimation of DE is central to the study of gene expression and, usually, the term is used to refer to a statistically significant difference in the experimentally determined abundance of an mRNA species between two treatments (7,8). We propose a generalized framework, which naturally extends to many target categories (e.g. transcripts, genes, genomic regions, etc.) with DE referring to any statistically significant response of interest. We demonstrate that the proposed methodology is robust and versatile in terms of handling small sample sizes and different experimental designs.

Given multiple probes per target, an intuitively appealing approach would be to treat probes as voters. As a result, each target can receive between 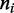 and 0 votes in favor of DE, where 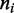 is the number of probes specific to target *i*. As 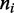 varies substantially, a practical measure of DE would be 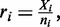 where 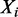 is the number of differentially expressed probes specific to target *i*. Testing for the alternative hypothesis 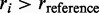 where 
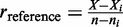
 with 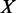 and 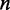 representing the total number of differentially expressed probes and the total number of probes on the array, respectively, involves the pooling of information from all probes to produce a target-specific estimate of DE. A test for the enrichment of target 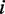-specific differentially expressed probes in relation to the reference can be formulated in terms of the hypergeometric distribution yielding the probability of the null-hypothesis

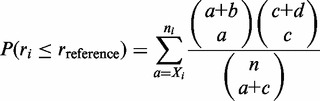

where *b = X* – *X_i_*, *c = n_i_* – *X_i_*, *d* = *n* – *n_i_* – (*X* – *X_i_*). The equation corresponds to the Fisher’s exact test with the alternative hypothesis 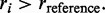


**Figure 1.**
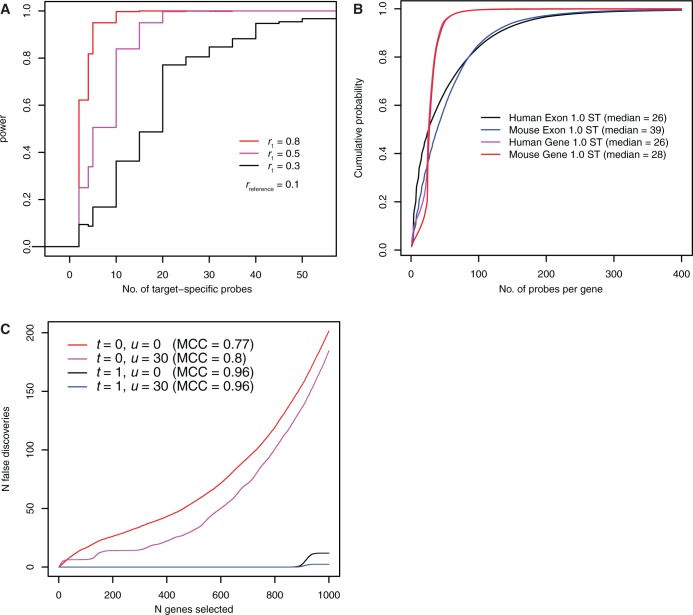
(**A**) Power analysis of Fisher’s exact test. For each combination of input parameters, the average of 1000 simulations is plotted. (**B**) Distribution of gene-specific probe counts. (**C**) Performance analysis based on DE estimates from simulated gene-expression data. For each combination of analysis parameters the average of 100 simulations is plotted.

In the present context, an important aspect of the test is that its power to reject the null hypothesis is dependent on 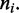 As demonstrated by power simulations based on realistic figures (Figure 1A), targets with 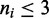 have an ∼60% chance of being detected as differentially expressed even if the enrichment is substantial (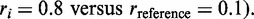 As the proportion of such targets on modern high-density microarrays is <5% (Figure 1B), it does not represent a major drawback for transcriptome analysis. On the other hand, the power to detect small differences in proportions (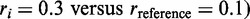 is high for 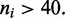 It might appear counterintuitive at first, but very high sensitivity can be potentially harmful as 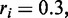 for example, contains substantially more evidence against the DE of target *i* even if significantly different from the reference rate. The question of a biologically meaningful difference between 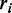 and 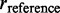 is related to the issue of meaningful fold-change (9,10) and there might not exist a single satisfactory solution. With that in mind, we propose an optional upper limit 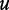 for 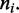 Censoring 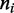 to 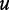 and adjusting 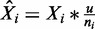 limits sensitivity when testing targets with *n_i_* > *u* thereby reducing the likelihood of falsely rejecting the null hypothesis. Based on our experience, a suitable value for *u* is ∼30, which is close to the median of gene-specific probes on high-density microarrays produced by Affymetrix. The fact that the significance of 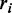 is determined relative to the background rate of differentially expressed probes, yields an interesting property of DEMI getting increasingly sensitive as the global signal profiles converge between samples. This feature is rather practical as the user is more likely to be interested in small differences when comparing inherently similar samples. Of note, as DEMI was designed specifically for estimating DE, currently, the accompanying software does not produce target-specific estimates of expression level.

## MATERIALS AND METHODS

### DEMI software

The accompanying software is available at http://biit.cs.ut.ee/demi.

### DEMI algorithm

DEMI is a probe-level framework to test for DE in microarray data. By design, it takes advantage of the fact that on modern high-density microarray platforms each target sequence is interrogated by many complementary probe sequences. The workflow consists of three-steps: (i) normalization of raw signal intensities, (ii) evaluation of probe-level signal dynamics and (iii) estimation of DE of the target in question. Normalization is necessary to render the signal distributions of individual arrays comparable and there are several ways to do it (e.g. ranking procedures, quantile normalization, etc.). Here, we have used relative ranking, because it provides an intuitive measure of gene expression by referring to its magnitude in relation to all other signals on the array (e.g. with 0 and 100 corresponding the weakest and strongest signals, respectively). In the second step, the evaluation of probe signal will establish whether the effect of the experimental treatment(s) is statistically significant on the probe level. Naturally, the choice of the test depends on the experimental design and the research question. In this study, for example, we have used Wilcoxon–Mann–Whitney test to establish DE between two experimental conditions and Kendall’s tau statistic to evaluate the departure of temporal expression dynamics from monotonicity. The final step, estimation of DE on the target level, is based on the hypergeometric probability for the enrichment of differentially expressed probes among target-specific probes (referred to as on-target probes) relative to the complement (i.e. off-target probes). Fundamentally, instances of different target categories such as exons, transcripts, genes and genomic regions are represented by proper subsets of the probes included on the array. Consequently, testing for the enrichment of differentially expressed probes among on-target probes as opposed to the off-target probes will correspond to the degree of DE of the target in question.

As an example of how the framework can be applied to the study of differential gene expression we present a non-parametric workflow identifying differentially expressed targets between a test sample (TEST) and a reference sample (REFERENCE). Given a dataset of *m* TEST and *n* REFERENCE individuals, each represented by an array of *q* independent gene-expression measurements (*m*, *n* > 0; *q* ≫ 0; *m*, *n*, *q* ∈ **N**) an input matrix 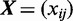 is constructed where 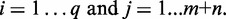 The rows of ***X*** correspond to independent measurements (equivalent to oligonucleotide probes on the array) and the columns correspond to individuals. In the first step, a normalizing transformation is applied column-wise to ***X*** whereby the signal distribution in each individual is normalized by converting the *q* signals into relative ranks
(1)
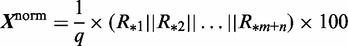

where 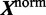 is the normalized input matrix, 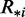 is a vector of ranks corresponding to column *i* of the input matrix and 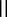 is the concatenation operator for column vectors. Relative ranking was chosen as the preferred method over other popular normalizing transformations such as quantile normalization and absolute ranking, because we find relative ranks to be intuitively easier to interpret and, hence, more meaningful to the end-user. Unlike quantile-normalized signals, the median relative rank is independent of the median signal intensity (it is always located ∼50) and, unlike absolute ranks, relative ranks are independent of the number of probes *q* (the normalized signal is always located between 0 and 100). For example, a relative rank of 25 indicates an expression level coinciding with the first quartile of the observed intensities.

In the second step, a statistical test is applied row-wise to 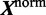 in order to classify probes as expressed higher or lower in TEST in relation to REFERENCE. Wilcoxon–Mann–Whitney rank sum test was chosen as the method, because it makes fewer assumptions about the signal distribution than *t*-test and it is less sensitive to outliers. Given the set of probes **Q** = {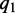, … ,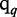}, vectors of equivalent normalized measurements 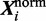 and the sets **M** = {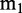, … 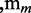}, **O** = {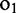, … 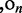} of column indices corresponding to TEST and REFERENCE individuals, respectively, we will define the set of up-regulated probes **H** and the set of down-regulated probes **L**. For *m, n >*3, the sets are defined as
(2)
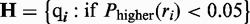

(3)
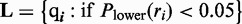

where 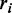 is a vector of ranks corresponding to row *i* of the normalized input matrix and 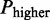 and 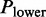 correspond to the h0 probability of obtaining a sum of ranks equal to and higher or lower, respectively, than the observed rank sum of TEST. In situations where 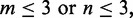 the following heuristic was used:
(4)
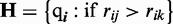

(5)
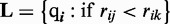

where 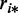 is a vector of ranks corresponding to row *i* of the normalized input matrix, 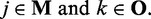 Accordingly, when one of the sample sizes was ≤3, a probe was labeled as differentially expressed only if all TEST ranks were either lower or higher than REFERENCE ranks. The heuristic is useful in situations where a *P*-value below 0.05 is theoretically unobtainable 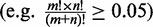 and a probe is to be classified as higher or lower in TEST if the sum of ranks in TEST individuals equals the maximum or the minimum, respectively.

Finally, in step 3, our aim is to identify the targets expressed higher or lower in TEST in relation to REFERENCE. Here, the targets refer to genes, transcripts or genomic regions. Following the core principle of oligonucleotide microarray design, the *t* targets (0 ≪ *t* ≪ *q*; *t* ∈ **N**) were related to the *q* independent expression measurements by a 100% identity between the nucleotide sequences of the probe and the target. Given a set of targets **T** = {t_1_, t_2_, … , t*_t_*} with target t*_i_* relating to a predetermined subset of probes 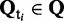 based on sequence identity and given sets of distinct probe expression profiles 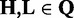 two complementary DE estimates can be obtained for each target. Since the situation can be conceptualized as a sampling without replacement problem, the hypergeometric probability distribution was used to obtain estimates under the null hypothesis of making |
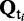
| draws randomly:
(6)
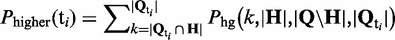

(7)
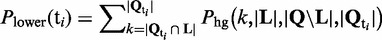

where *P*_hg_ is the hypergeometric distribution function. When targets are exons in the context of a gene, the formulas above are modified to yield the following:
(8)
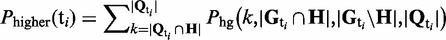

(9)
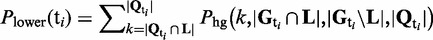

where 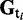 is the set of probes targeting the gene corresponding to target 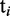. Of note, there is no need to calculate 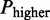 for 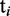 if 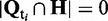 and the same applies for 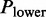 if 
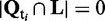
 as the result is by Definition (1). If **Q** is taken to represent the set of genes targeted by the array with **H** and **L** being the up- and down-regulated genes, respectively, and **T** is a collection of gene ontologies each represented by a gene set 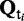 then Equations (6) and (7) yield gene ontology (GO)-level DE estimates. This methodology is well known and it belongs among the Class 1 gene-category tests defined in (11) which have been criticized for the increased incidence of Type I errors due to correlations between gene-expression profiles (12,13). However, this problem can be ameliorated by using a more stringent FDR procedure, which is the solution opted below. Furthermore, in the context of functional annotation analysis, Type II errors have much more serious consequences (e.g. failure to detect a differentially expressed pathway) than labeling a fraction of negatives as positives.

After adjusting the *P*-values for multiple hypotheses testing by the FDR procedure (14), the resulting estimates <0.05 were considered statistically significant. For target categories where a substantial overlap of targets in terms of on-target probes is anticipated (transcripts, genomic regions and functional categories) a modified version of the FDR procedure under dependency (15) is used to control Type I error rate.

### Microarray and Taqman® datasets

Datasets based on MicroArray Quality Control (MAQC) project’s samples of Human Brain Reference RNA, HBR (Ambion) and Universal Human Reference RNA, UHR (Stratagene) were obtained for the following platforms: GeneChip® Human Genome U133 Plus 2.0 (Affymetrix), GeneChip® Human Gene 1.0 ST Array (Affymetrix), GeneChip® Human Exon 1.0 ST (Affymetrix), Taqman® (Applied Biosystems) (Table 1). Four technical replicates were used for the subsequent analysis in all platforms.

**Table 1.** Microarray and Taqman® datasets representing MAQC reference samples in this article

Platform	Accession	Reference
Human Genome U133 Plus 2.0	GEO:GSE9819	(16)
Human Gene 1.0 ST	GEO:GSE9819	(16)
Human Exon 1.0 ST	GEO:GSE13069	(17)
Taqman®	GEO:GSE5350	(18)

## Analysis of RNA-seq datasets

Two different RNA-seq datasets (Table 2) based on HBR and UHR samples were obtained from the NCBI’s Gene Expression Omnibus database (GSE12946, GSE24283). The reads were mapped with TopHat (19) (version 1.3.3) that uses Bowtie (20) (version 0.12.7) to obtain the alignments. Annotation information and the indices were built on the data downloaded from the Ensembl database (build GRCh37.p12). The mapped reads were used to estimate differential gene expression between HBR and UHR samples using three state-of-the-art methods: EdgeR (21) (R package, version 3.0.8), DESeq (22) (R package, version 1.10.1) and Cuffdiff2 (23) (stand-alone, version 2.0.2). EdgeR and DESeq estimates were based on gene-level read counts produced by HTSeq (http://www-huber.embl.de/users/anders/HTSeq/) version 0.5.3p9, using flags ‘–stranded=no –mode=union –type=exon’.

**Table 2.** Number of spots per sample for RNA-seq datasets representing MAQC reference samples in this article

Dataset	HBR	UHR
GEO:GSE12946	17246957	8069964
GEO:GSE24283	53238798	59461348

### Sequence retrieval and annotation

Probe sequences of Affymetrix gene-expression arrays were downloaded from the company’s website (www.affymetrix.com). Genome and transcriptome sequences corresponding to release 73 of Ensembl were downloaded from Ensembl’s public FTP site (ftp.ensembl.org), annotation tables for matching identifiers were downloaded from Ensembl’s BioMart using biomaRt package (http://www.bioconductor.org/packages/release/bioc/html/biomaRt.html).

Genomic karyotype information was obtained from Ensembl databases using bioperl-1.2.3 (http://www.bioperl.org) and Ensembl Perl API (http://www.ensembl.org/info/data/api.html).

### Probe sequence alignment

Probe sequences were aligned to the genome, transcriptome and exome using the Blat application (24). An un-gapped alignment of at least 23 nucleotides was required to produce a hit. Probe annotations are summarized in Supplementary Table S1.

### Processing of microarray data

To compare DEMI with state-of-the-art workflows for DE analysis four different probe set summarization methods were used: RMA (25), FARMS (26), DFW (27) and PLIER (http://media.affymetrix.com/support/technical/technotes/plier_technote.pdf). RMA, FARMS and DFW were applied using the implementation provided in the xps package (http://www.bioconductor.org/packages/devel/bioc/html/xps.html) and PLIER was applied using Affymetrix Power Tools (http://www.affymetrix.com/estore/partners_programs/programs/developer/tools/powertools.affx). All methods were applied with default parameters. Probe sets were mapped to the corresponding Ensembl gene ID’s based on annotation data downloaded from Ensembl’s BioMart.

### DE estimates from Taqman® assays in MAQC data

Gene-expression data from Taqman® assays pertaining to Human Brain Reference RNA and Universal Human Reference RNA samples was retrieved from Gene Expression Omnibus (GEO:GSE5350). In the dataset, normalized gene expression levels were obtained by the MAQC consortium using the formula 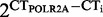 where CT_i_ refers to the cycle threshold of the gene of interest and POLR2A gene is the reference. We estimated DE between the two RNA samples by comparing normalized expression values from replicate assays using Student’s *t*-test followed by Bonferroni correction using R software (www.r-project.org). Genes with adjusted *P*-value < 0.05 were labeled as differentially expressed. The list of differentially expressed genes based on Taqman assays was used as the reference during benchmarking.

### Performance evaluation of DE estimation in MAQC data

DE estimates from microarray data were obtained by Limma (28), RankProd (29,30) and DEMI while the estimates from Taqman® assays were used as the reference when evaluating performance. The number of true positives (TP), false positives (FP), true negatives (TN) and false negatives (FN) were calculated on the intersection of gene identifiers in the prediction and reference datasets. A prediction was labeled as TP if the corresponding adjusted *P*-values were significant in both datasets and there was an agreement in the direction of DE (i.e. higher/lower expression in the Human Brain Reference RNA when compared to Universal Human Reference RNA in both datasets). A prediction was labeled TN if the corresponding adjusted *P*-values were insignificant in both datasets. A prediction was labeled FP if the corresponding adjusted *P*-value was significant in the prediction dataset, but not in the reference dataset. Finally, a prediction was labeled FN if the corresponding adjusted *P*-value was insignificant in the prediction dataset, but significant in the reference dataset. The number of true positives, false positives, true negatives, false negatives and the MCC were calculated using the ROCR package in R (http://cran.r-project.org/web/packages/ROCR/).

### Analysis of array permutations

For comprehensive testing of performance on sample sizes 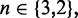 all *m* by *n* permutations of the *m* = 4 original arrays were created. Each permutation of the test sample was compared against all permutations of the reference. For each comparison, the performance indicators were obtained by calculating the mean value across the selected FDR cutoffs. The performance indicator for each analysis workflow was obtained by averaging across all comparisons. Significance analysis of MCC values obtained for various workflows under identical conditions was performed using paired *t*-test.

### Retrieval of GO information

The child categories of all GO terms were retrieved using Ensembl Perl API. Ensembl gene identifiers corresponding to each GO category were downloaded using biomaRt package providing programmatic access to the Ensembl’s Biomart database. Gene list corresponding to a GO category was compiled from gene identifiers associated with the category and its children.

### Analysis of long-range epigenetic silencing data

The dataset (GEO:GSE19726) contained two replicate gene-expression measurements by Human Gene 1.0 ST array (Affymetrix) of normal prostate epithelial cells (PrEC) and the prostate cancer cell line LNCaP. A list of putative candidate regions subject to long-range epigenetic silencing (LRES) in prostate cancer was obtained from Table 1 of the original study (31). DEMI was used to estimate DE in 0.5-Mbp genomic windows overlapping by 50%. The original comparison and the two null permutations of the arrays were analyzed. Due to overlap in the genomic windows, *P*-values were adjusted for multiple testing using the method by Benjamini and Yekutieli (15), a default setting in DEMI when genome is the target. Adjusted *P*-values < 0.05 were considered statistically significant. Minimally, a 0.25-Mbp overlap between a down-regulated genomic window and a candidate LRES locus was required to label the LRES locus as detected by DEMI.

### Enrichment analysis of differential epigenetic modification

The ChIP-chip data from (31) (MAT scores from two arrays per cell line and histone modification type) representing H3K9 acetylation and H3K27 tri-methylation in LNCaP and PrEC cells were downloaded from Gene Expression Omnibus (accession no. GSE19726). Before statistical testing, the MAT scores were quantile normalized in R (package preprocessCore) to ensure comparable signal distributions between the arrays. Differential chromatin modification was estimated in the same genomic regions as analysed for DE by DEMI. All probes mapping to the genomic region of interest were included in the analysis. Differential chromatin modification of a genomic region was estimated using probe-wise paired *t*-test between LNCaP and PrEC arrays followed by Bonferroni correction. Enrichment of regions exhibiting differential chromatin modification was estimated among differentially expressed genomic regions using the hypergeometric probability distribution.

### Primary cell culture model of hypoxia

Around 1 million primary mouse embryonic fibroblasts (Millipore) were seeded onto 100-mm culture dishes and were grown in DMEM (high glucose 4.5 g/l, supplemented with 10% FBS and L-glutamine, PAA) in normal conditions (atmospheric oxygen, 5% CO_2_ at 37°C) until 60–70% confluent. Hypoxia was initiated by lowering the oxygen concentration to 1% in a multi-gas incubator (Sanyo). The experiment was carried out in five biological replicates per experimental condition. After 24 h, RNA was extracted from the hypoxic and normoxic cells by Trizol® (Life) followed by large scale gene expression profiling with Mouse Exon 1.0 ST array (Affymetrix) according to manufacturer’s protocols. Briefly, 50 ng of total RNA from each sample was amplified using the Ovation Pico WTA system V2 (Nugene). Fragmentation and biotin labeling was done using the Encore-Ovation cDNA Biotin Module (Nugene). The labeled samples were hybridized to the Mouse Exon 1.0 ST array (Affymetrix). The arrays were washed and stained with phycoerytrin conjugated streptavidin (SAPE) using the Affymetrix Fluidics Station® 450, and the arrays were scanned in the Affymetrix GeneArray® 3000 scanner to generate fluorescent images, as described in the Affymetrix GeneChip® protocol. Cell-intensity (CEL) files were generated in the GeneChip® Command Console® Software (AGCC) (Affymetrix).

### Primary cell-culture model of hypothermia

Around 1 million primary mouse embryonic fibroblasts (Millipore) were seeded onto 100-mm culture dishes and were grown in DMEM (high glucose 4.5 g/l, supplemented with 10% FBS and L-glutamine, PAA) in normal conditions (atmospheric oxygen, 5% CO_2_ at 37°C) until 60–70% confluent. Hypothermia was initiated by lowering the temperature of the cell culture incubator to 32°C. The control arm of the experiment was incubated at 37°C. Three dishes per group were incubated for various durations (0, 0.5, 1, 2, 4, 8, 18 h) followed by extraction of RNA with Trizol (Life). Pooled RNA from the three replicates was subjected to large-scale gene-expression profiling on the Mouse Gene 1.0 ST array (Affymetrix) according to manufacturer’s protocols. RNA was amplified and labeled using the Ambion WT Expression Kit (Applied Biosystems) according to manufactures instructions. As input, 250 ng total RNA was used. The labeled samples were hybridized to the Mouse Gene 1.0 ST GeneChip® array (Affymetrix). The arrays were washed and stained with phycoerytrin conjugated streptavidin (SAPE) using the Affymetrix Fluidics Station® 450, and the arrays were scanned in the Affymetrix GeneArray® 3000 scanner to generate fluorescent images, as described in the Affymetrix GeneChip® protocol. CEL files were generated in the GeneChip® Command Console® Software (AGCC) (Affymetrix).

### Quantitative real-time PCR

Total RNA was extracted from cells using Trizol® Reagent (Invitrogen, USA) according to the manufacturer’s protocol. First-strand cDNA was synthesized with random hexamers (Invitrogen, USA) and SuperScript™ III Reverse Transcriptase (Invitrogen, USA). The following Taqman® (Applied Biosystems) assays with FAM-labeled probes were used in the study: Mm00483336_g1 (Cirbp), Mm01253561_m1 (Nqo1), Mm00515065_m1 (Gss), Mm00443675_m1 (Txnrd1), Mm01609819_g1 (Rbm3), Mm00802655_m1 (Gclc), Mm00769566_m1 (Srxn1), Mm01281449_m1 (Vegfa). Assay Mm01158416_g1 (Ywhaz) with VIC-labeled probe was used as reference. qPCR reactions were run on the ABI PRISM 7900HT Fast Real-Time PCR System equipment (PE Applied Biosystems, USA) and quantified with the ABI PRISM 7900 SDS 2.2.2 software. For each assay, an average of four technical replicates was used as the endpoint.

### Enrichment analysis of HIF-1- and HIF-2-binding sites in hypoxia-induced genes

Lists of high-stringency HIF-1- and HIF-2-binding sites were obtained from the Supplementary Material of Schödel *et al.* (32). Annotation table linking human gene identifiers (RefSeq) to corresponding mouse orthologs (Ensembl) was downloaded from Ensembl using R (package biomaRt). As some of the human gene identifiers did not map to a mouse orthologue we were able to identify 295 mouse orthologs of HIF-1 targets and 245 orthologs of HIF-2 targets. Enrichment of putative Hif-1 and Hif-2 target genes was estimated among significantly up-regulated genes using the hypergeometric probability distribution.

## RESULTS

### Computer simulations

To test the reasoning above, we performed 100 simulations of microarray experiments with two groups (*N* = 4) involving 45 000 genes and around 1.3 million probes. Up- and down-regulation of randomly picked 1000 genes was simulated by adding a fold-change of 2 or −2 to the log_2_-transformed intensities of 80% of target-specific probes 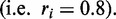 Noise was added by applying the same fold-change to a randomly chosen 10% of the remaining probes 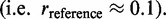 False discovery rates and Matthews correlation coefficient (MCC) were studied after adjusting two limiting values, *u* (the maximum number of probes matching to a target) and *t* (the maximum number of distinct targets matching to a single probe). In the baseline setting, both *u* and *t* were not in effect. Setting *t* to 1 caused a considerable reduction in the number of false positives, which was expected as differentially expressed probes mapping to multiple targets affect multiple 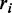 − s (Figure 1C). Setting *u* to 30 had a less pronounced albeit still beneficial effect on the false positive rate (FPR). On average, only two false positives were found when both of the parameters were in effect. Taken together, the simulations suggest that on artificial data DEMI produces highly accurate results when *t* = 1 and *u* = 30.

### MAQC data

To benchmark DEMI in relation to established methods of high-throughput gene-expression analysis, the most comprehensively characterized dataset, the MAQC reference samples (18), was studied. The dataset included three microarray platforms (Table 1) and high-throughput RNA-sequencing data (RNA-seq) from independent sources (Table 2). Microarray data was analyzed by DEMI and eight conventional DE analysis workflows. Performance was evaluated at different sample sizes (*N* = {4 … 2}) using numerous well-established metrics including area under receiver-operator curve (AUC), MCC, true positive rate (TPR), FPR, true negative rate (TNR) and false negative rate (FNR). Cutoff-dependent metrics (MCC, TPR, FPR, TNR, FNR) were summarized as the average of two FDR cutoffs (0.05, 0.01). For comparison, two RNA-seq datasets representing the MAQC samples were analyzed by three state-of-the-art methods (Cuffdiff 2.0, DESeq, edgeR).

**Figure 2.**
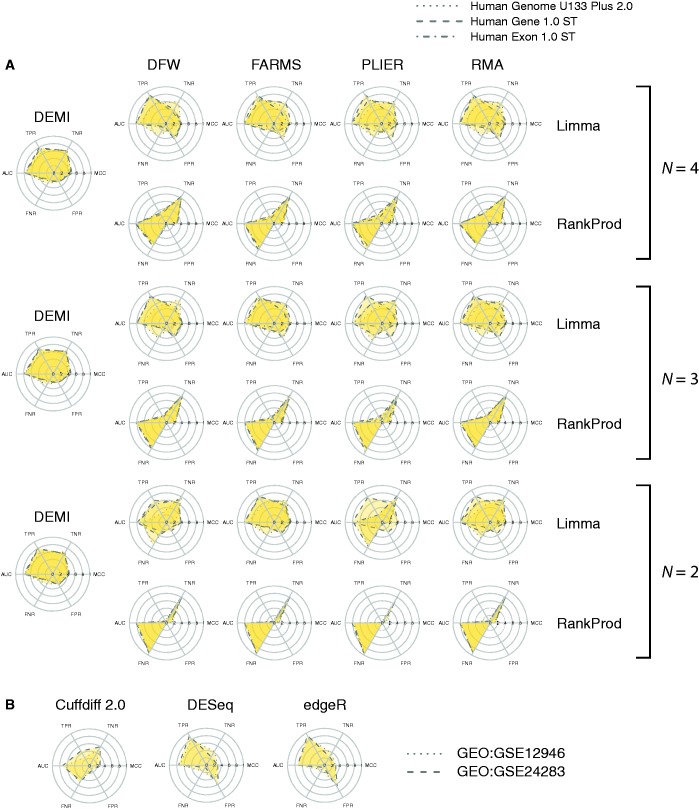
Performance analysis of DE analysis pipelines on the MAQC data. Performance of each pipeline is presented as a radial plot, which includes results from three microarrays (**A**) or two distinct RNA-seq datasets (**B**) based on six complementary performance indicators. Abbreviations: AUC, MCC, TPR, FPR, TNR and FNR.

To offer a better overview of the benchmarking endpoints, appropriate performance indicators were juxtaposed on radial plots, which we refer to as ‘balanced performance plots’ (Figure 2). Perfect performance is represented on these plots by a colored plane occupying most of the upper semicircle while the lower semicircle remains blank. The benchmarking results are also available in numerical form (Supplementary Material, file 2). Overall, DEMI exhibited most stable performance across the tested microarrays and sample sizes (Figure 2A). RankProd appeared to lack power at the given FDR cutoffs as indicated by a relatively lower TPR, higher FNR and a larger difference between AUC and MCC. Limma was the most sensitive method at *N* = {4, 3} in terms of the TPR, but it exhibited a relatively higher FPR and, consequently, similar MCC to DEMI. Performance plots of PLIER appeared less consistent between the arrays than was the case for the other normalization methods. Significance analysis of MCC values obtained for various workflows indicated small, but mostly significant differences under otherwise identical conditions (Supplementary Material, file 3). We conclude that the performance of DEMI is comparable to the best-performing workflows, but it is more stable across arrays and sample sizes. Benchmarking of the RNA-seq data analysis pipelines revealed somewhat lower MCC in relation to the best-performing microarray analysis methods (Figure 2B). While DESeq and edgeR displayed higher TPR than array analysis methods, it came at the expence of a notably higher FPR resulting in MCC values ∼0.3. Cuffdiff 2.0 was the most conservative of the three with substantially lower TPR, a very low FPR and an MCC slightly inferior to the other RNA-seq analysis methods.

### Cell culture model of hypoxia

**Figure 3.**
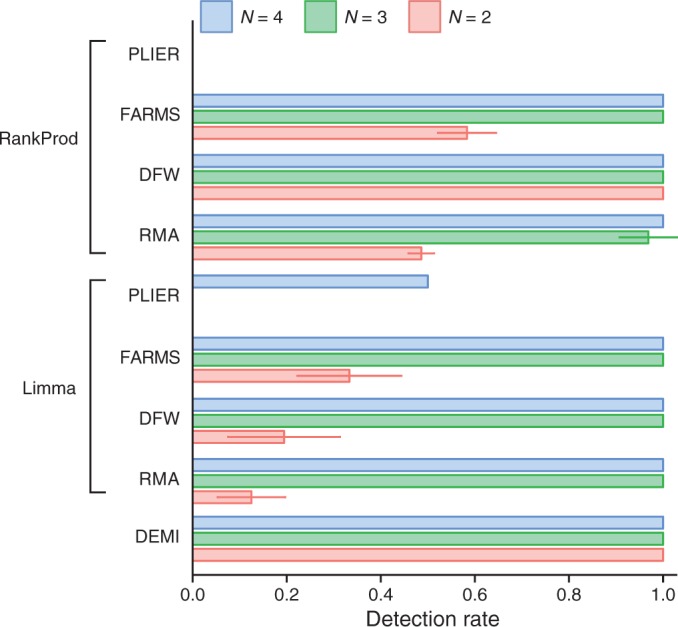
Performance analysis of DE-analysis pipelines on data from mouse embryonic fibroblasts exposed to 1% O_2_ for 24 h. The plot depicts the combined detection rate of GO categories ‘cellular response to hypoxia’ (GO:0071456) and ‘glycolysis’ (GO:0006096) as indicators of hypoxia response. The data is plotted as mean ± standard error of all possible comparisons between subsets of size *N* of the hypoxic and normoxic groups (original *N* = 4).

In order to benchmark DEMI in experimental settings, we sought to set up and validate a novel *in vitro* model of hypoxia using a primary cell culture of mouse embryonic fibroblasts. The fact that the transcriptional mechanisms of hypoxia response have been extensively studied enabled us to validate the cell model and the analysis methodologies in relation to established knowledge in the field (33,34). The cells were subjected either to 24 h of 1% O_2_ or atmospheric oxygen followed by large-scale gene-expression profiling using the Mouse Exon 1.0 ST array (Affymetrix). Differential gene-expression estimates from eight workflows were submitted to functional category analysis in g:Profiler (35) while the built-in analysis was used in DEMI (Supplementary Material, files 4–5). We looked for the up-regulation of the GO categories ‘cellular response to hypoxia’ (GO:0071456) and ‘glycolysis’ (GO:0006096) as indicators of the hypoxia response (36). Nearly all workflows displayed perfect pathway detection rate when 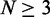 whereas only DEMI had perfect detection rate at 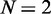 indicating that both pathways were detected as significantly upregulated in all 2-element subsets of the original samples with *N* = 4 (Figure 3). To provide an additional measure of accuracy the enrichment of mouse orthologs of HIF-1 targets among hypoxia-induced genes was studied (Table 3). Most methods produced a highly significant enrichment of putative Hif-1 target genes among up-regulated genes when *N* ≥ 3. DEMI was least affected by the reduction in sample size as indicated by a robust enrichment of putative Hif1-target genes when *N* = 2. Essentially similar results were obtained for putative Hif-2 targets (Supplementary Table S2).

**Table 3.** Enrichment of mouse orthologs of HIF-1 targets among significantly up-regulated genes in mouse embryonic fibroblasts exposed to 1% O_2_ for 24 h

Normalization	DE	*N* = 4		*N* = 3		*N* = 2	
		Mean	SEM	Mean	SEM	Mean	SEM
Relative ranking	DEMI	3.0*E*-25	NA	4.3*E*-22	2.4*E*-22	6.0*E*-19	5.4*E*-19
DFW	Limma	1.4*E*-28	NA	3.5*E*-23	2.2*E*-23	0.617	0.081
DFW	RankProd	1.4*E*-30	NA	2.2*E*-23	1.5*E*-23	8.2*E*-08	4.8*E*-08
FARMS	Limma	9.5*E*-22	NA	2.5*E*-21	1.9*E*-21	0.457	0.082
FARMS	RankProd	6.8*E*-20	NA	3.1*E*-10	2.1*E*-10	0.037	0.011
PLIER	Limma	9.4*E*-10	NA	0.9	0.1	1	0
PLIER	RankProd	3.5*E*-01	NA	1	0	1	0
RMA	Limma	6.0*E*-19	NA	1.7*E*-14	1.5*E*-14	0.481	0.083
RMA	RankProd	1.4*E*-18	NA	4.3*E*-13	2.1*E*-13	0.001	4.6*E*-04

Differential gene expression was estimated by nine pipelines including various normalization and DE-analysis methods. The data is presented as mean and standard error of hypergeometric *P*-values from all possible comparisons between subsets of size *N* of the hypoxic and normoxic groups (original *N* = 4). *N*, sample size; SEM, standard error of mean; NA, not available.

### LRES

To demonstrate that DEMI can evaluate the expression of unconventional target categories, such as genomic regions, it was applied to gene-expression data related to LRES. LRES refers to the suppression of gene expression from large chromosomal regions and it might be related to cancer progression (37). The original study used several independent lines of evidence to identify putative epigenetically silenced genomic regions in prostate cancer (31). Here, we used DEMI to identify down-regulated genomic regions in prostate cancer cell line LNCaP in relation to normal prostate epithelial cells from two replicate gene-expression array measurements per cell line as provided by the original study. DEMI identified 2242 of the 22 630 genomic regions (each region spanning 0.5 Mbp) as down regulated (Supplementary Material, file 6). In array permutations corresponding to the null hypothesis the fraction was an order of magnitude smaller (222 and 201). In total, 38 out of the 47 putative LRES loci identified in the original study were found to be significantly down-regulated (81%). Furthermore, the enrichment of putative LRES loci among down-regulated regions was highly significant (*p* = 3.04*e*-13, Fisher's Exact Test). The enrichment was not apparent in array permutations corresponding to the null hypothesis (*p*_P1_ = 0.961; *p*_P2_ = 1, Fisher’s Exact Test). To confirm that differentially expressed genomic regions are associated with altered chromatin modification between LNCaP and PrEC cells we compared the levels of H3K27 tri-methylation and H3K9 acetylation based on ChIP-chip data from the original study. Similarly to the original study it was confirmed that the down-regulation of H3K9ac is associated with silenced genomic regions (*p* = 5.400*E*-22, hypergeometric probability distribution) while the up-regulation of H3K9ac is prevalent among up-regulated regions (*p* = 7.990*E*-05, hypergeometric probability distribution) in LNCaP cells when compared to PrEC (Table 4). No significant association between H3K27me3 and DE was found. Taken together, present results indicate that DEMI accurately identifies genomic regions that are candidates for long-range epigenetic modification of gene expression.

**Table 4.** Enrichment of regions exhibiting differential epigenetic modification among differentially expressed genomic regions between LNCaP and PrEC cell lines

	Higher modification level in LNCaP	Lower modification level in LNCaP	Modification
Higher expression level in LNCaP	0.998	0.266	H3K27me3
	7.990*E*-05	1	H3K9ac
Lower expression level in LNCaP	0.102	1	H3K27me3
	1	5.400*E*-22	H3K9ac

Significant *P*-values (hypergeometric probability distribution) indicate that down-regulation of H3K9ac is associated with silenced genomic regions while up-regulation of H3K9ac is prevalent among up-regulated regions in LNCaP cells when compared to PrEC. No significant association was found between DE and H3K27me3.

### Cell culture model of hypothermia

**Figure 4.**
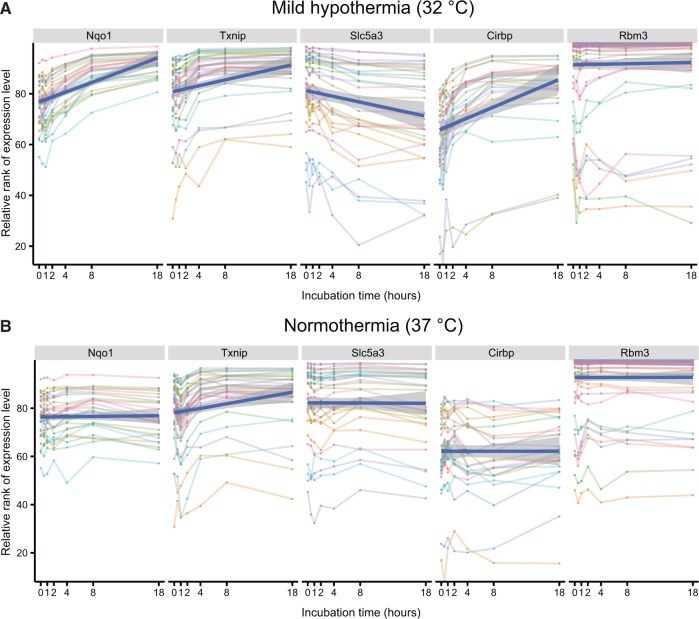
Large-scale analysis of temporal dynamics of transcription in mouse embryonic fibroblasts exposed to mild hypothermia. (**A** and **B**) Temporal profiles of probe expression levels of selected genes during mild hypothermia (**A**) and normothermia (**B**). The solid blue line indicates a linear fit to the data points and the gray shadowing represents standard error of the fit.

**Figure 5.**
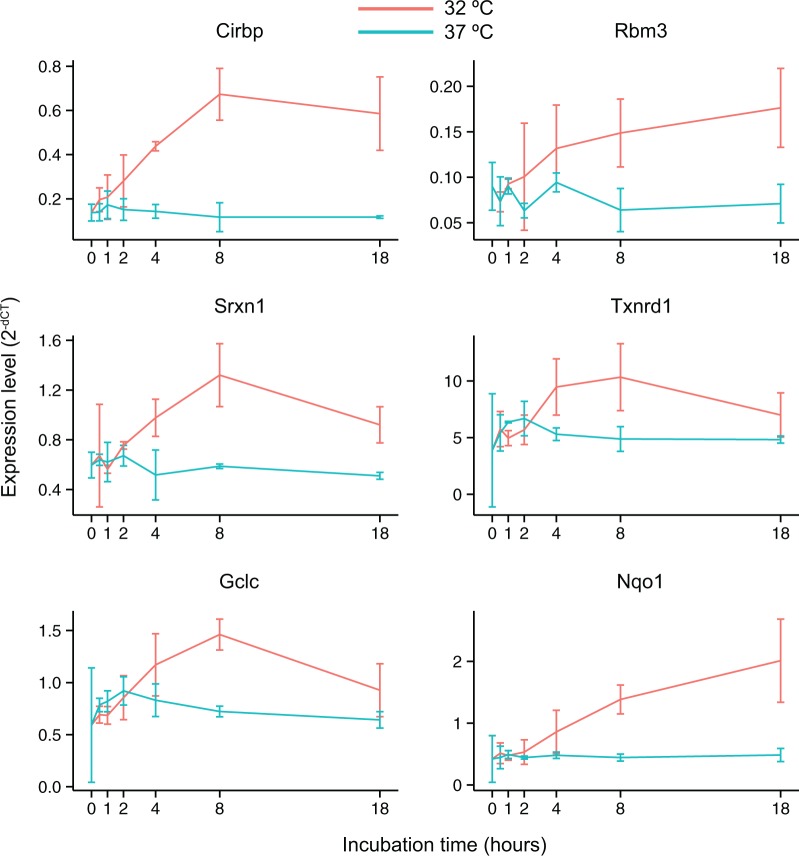
Temporal expression profiles of selected genes during mild hypothermia (32°C) and normothermia (37°C) as reported by quantitative real-time PCR. Expression level of *Ywhaz* was used as reference. The mean of three replicates and standard error has been plotted.

Finally, we devised a strategy to demonstrate that DEMI can handle novel experimental designs unrelated to group-wise comparisons. Therapeutic hypothermia is a clinically effective treatment for various hypoxic and ischemic conditions (38–40), but the associated molecular mechanisms remain unclear. To gain insight into hypothermia-induced transcriptional response, mouse embryonic fibroblasts were exposed to mild hypothermia (32°C) or normothermia (37°C) for increasing time periods. We aimed to identify genes with temporally near-monotonic response as the most obvious candidates for mediating the therapeutic effects of hypothermia. Monotonicity is characteristic of many physiological responses (including the activation of transcription) as demonstrated by the wide applicability of the Michaelis–Menten kinetics and the Hill equation, which describe nonlinear and saturable mechanisms (41). We reasoned that a near-monotonic temporal relation between gene expression and hypothermia is a more selective indicator of a causal relation than the comparison of treatment to control at any single point in time. Importantly, the experimental design required only 13 arrays to study hypothermic and normothermic response at seven time points. The departure of probe-level responses from monotonicity was evaluated using Kendall’s tau statistic, a measure of rank correlation. We identified 1750 and 274 genes with significantly monotonic-like temporal response to hypothermia and normothermia, respectively (Supplementary Material, files 7 and 8). The previously established hypothermia-responsive gene Cold inducible RNA-binding protein, *Cirbp* (42,43) was found among the top-five of up-regulated genes with 24 probes out of 25 exhibiting a statistically significant monotonic-like response (Figure 4A). In contrast, none of the *Cirbp*-specific probes displayed a significant response when incubated at 37°C (Figure 4B) validating both the experimental design and our analysis strategy. As a novel insight, we identified time-dependent increase in the expression of multiple genes related to the two major antioxidant pathways, the glutathione and thioredoxin systems (Table 5). Results from the microarray study were confirmed by qPCR (Figure 5). To our knowledge, this is the first study to report coordinated up-regulation of antioxidant gene expression by hypothermia.

**Table 5.** Genes responding to increasing durations of hypothermia with significantly monotonic increase in expression and relating to the antioxidant system

Gene ID	Symbol	*P*-value	FDR	System
ENSMUSG00000003849	Nqo1	9.04*E*-35	4.72*E*-30	Quinone detoxification
ENSMUSG00000032802	Srxn1	1.23*E*-19	2.14*E*-16	Glutathione
ENSMUSG00000027610	Gss	1.08*E*-13	4.74*E*-11	Glutathione
ENSMUSG00000020250	Txnrd1	4.29*E*-11	9.83*E*-09	Thioredoxin
ENSMUSG00000032350	Gclc	4.29*E*-11	9.83*E*-09	Glutathione
ENSMUSG00000000811	Txnrd3	9.85*E*-09	1.19*E*-06	Thioredoxin

## DISCUSSION

DEMI makes a notable departure from the conventional methods of DE analysis (5,6,44) in a couple of respects. First, a variety of statistical tests can be used to evaluate DE on probe level. Such flexibility can be used to adapt the method to a variety of experimental designs. Here we have demonstrated the use of Wilcoxon–Mann–Whitney test and Kendall’s tau to calculate probe-level statistics for two-group comparisons and time-series, respectively. Similarly, it should be possible to adapt DEMI to factorial designs (using probe-level ANOVA) and to identifying relationships between covariates (ANCOVA), for example. Second, DEMI summarizes probe-level responses after testing for DE, whereas conventionally, probe signals are summarized before testing. Our results suggest that having more data points at disposal enables more robust evaluation of DE by compensating for the loss of statistical power when *N* is very small. Finally, it seems likely that DEMI can be adapted to the analysis of RNA sequencing and proteomic data as well. Fundamentally, both RNA-seq and proteomic analysis of tryptic peptides estimate the abundance of the target by concurrent quantification of its numerous fragments. As there are a number of practical issues, which need to be addressed, a thorough discussion is currently out of scope.

In the present article, several unrelated datasets were used to evaluate the performance of DEMI. The datasets were chosen carefully to include different application domains (e.g. gene versus genomic region-expression analysis), experimental designs (comparison of two groups versus time-series experiment), and to enable comprehensive bechmarking (MAQC data, evaluation of hypoxia response). First, twelve DE estimation workflows were benchmarked on the MAQC reference samples including data from three microarray platforms and two RNAseq studies. While it can be argued that the average gene expression study might not be faithfully represented by the MAQC reference samples (differences between the Human Brain RNA and Universal Human RNA samples appear to be uncommonly large and there is a lack of biological variation between replicates) the fact that the samples have been thoroughly characterized by various gene-expression assays makes it an attractive dataset for benchmarking. In the present article, an indicator of major differences between the MAQC reference samples was the considerably higher rate of differentially expressed probes in the MAQC data when compared to the tissue culture model of hypoxia (38% in MAQC samples versus 11% in hypoxia). Our argument is substantiated by the consensus that hypoxia exerts wide-spread effects on gene expression (36,45).

In order to evaluate DE workflows in a more natural context, we devised and conducted an experiment comparing the transcriptomes of hypoxic and normoxic mouse embryonic fibroblasts. Our choice was motivated by the facts that the molecular mechanisms of hypoxia response have been extensively characterized and relevant pathways are included in the GO database. Accordingly, we were able to evaluate the accuracy of pathway-level DE predictions based on the detection rate of pathways linked to the experimental response by definition. We believe that this approach has high validity and it should be preferred over computer simulations, which are unlikely to capture the probe- and target-level correlations arising from biological and technical causes in natural datasets (12,46,47).

Next, the dataset of Coolen *et al.* (31) was used to demonstrate the efficacy of DEMI in detecting DE on the genomic level, a level of analysis rarely approached by traditional DE workflows. As epigenetic modification of chromatin can have widespread effects on DNA expression, we expect this type of analysis to be of great relevance. Finally, we devised and validated a novel method for detecting temporally near-monotonic expression patterns to demonstrate that DEMI is extendable beyond two-group comparisons. In consequence, we identified hypothermia-dependent up-regulation of genes encoding for major constituents of the antioxidant response as a possibly novel route for the therapeutic effects of hypothermia.

As endorsed by the MAQC consortium (48), we used MCC (49) as the indicator for benchmarking performance on the MAQC reference samples, because it is informative when the distribution of the two classes in a dataset is skewed. Based on the Taqman® assays, the numbers of positive and negative findings was 569 and 298, respectively, substantiating the choice of MCC as a primary performance indicator. For an even more comprehensive view, a number of related performance indicators, such as AUC, TPR, FPR, TNR and FNR, were included in a single radial plot we termed as a balanced performance plot. Although related, MCC and AUC represent different approaches to performance as the former is cutoff-dependent while the latter is not. As statistical reasoning is usually based on a predetermined significance level (e.g. *P* < 0.05), there are potentially many cases where AUC might not yield meaningful results. For example, given vector 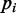 (*i* = 1 … *n*) of *n P*-values the AUC is calculated by integrating the ROC curve over the range of 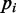 whereas in the context of statistical testing cutoff 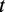 is used for rejecting the null hypothesis. As AUC is cutoff-independent, an estimator with 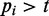 can still yield excellent AUC values even though TPR and FPR at 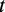 are 0 in which case MCC is 0. Using MCC and AUC simultaneously has the benefit of identifying cases where the predictions are accurate (based on AUC), but the method lacks statistical power at the given cutoff (indicated by MCC) as was the case with RankProd in the current study.

In parallel with the increasing popularity of high-throughput sequencing platforms, there have been numerous claims that studying gene expression by RNA-seq technology is superior to microarray analysis (23,50–53). Of the aforementioned publications, two studies (23,50) compared the performance of RNA-seq and microarrays in terms of DE-detection accuracy. For a number of reasons, the studies do not provide substantial evidence for the increased accuracy of RNA-seq-based estimates. For example, the dataset used by Marioni *et al.* (50) lacked extensive quantitative PCR (qPCR) data (expression levels of only 11 genes were available) making it clearly inferior to the MAQC dataset used here. Second, an outdated array platform (HG-U133 Plus 2.0, Affymetrix) was used with approximately five-times lower coverage of targets in terms of total probe hits than the Human Exon 1.0 ST array. Most importantly, neither AUC nor MCC was used as a performance indicator in the study. The more recent study by Trapnell *et al.* (23) used the MAQC data, but the performance of RNA-seq based estimates was not evaluated in relation to the available microarray data. In addition, the performance was evaluated against fold-change estimates from the qPCR whereby genes with a log_2_-fold change of >2.0 were declared DE. Such practice has been deemed unsatisfactory by the microarray community due to fold-change not being an inferential statistic as it does not produce known and controllable long-range error rates (44).

On the other hand, we are aware of at least two studies suggesting that high-sequencing depth is critical for differential analysis of transcripts present at low abundance (2,23). Accordingly, a comparison of RNA-seq with a high-density microarray platform indicated higher sensitivity of the latter in detecting DE for low abundance genes (2). Similarly, several studies have reported a high variation (i.e. unreliable detection) in the expression level of targets with low read counts (1,50,54). Furthermore, DE-detection sensitivity is lower for shorter exons when compared to the microarray (55). These biases are consistent with a uniform sampling hypothesis whereby less abundant and shorter nucleic acid molecules yield fewer fragments and, consequently, less reads to base the estimates on. To illustrate, Łabaj *et al.* (54) have estimated that 75% of the RNA-seq’s measurement power is spent on only 7% of the known transcriptome. In addition, it was estimated that ∼30% of the transcriptome might exhibit read counts too low for accurate prediction of DE. Our extensive analysis including three microarray platforms, >10 analysis pipelines and various sample sizes indicate that the accuracy of DE estimates obtained from the RNA-seq data available on the MAQC reference samples is slightly lower than for the tested microarray platforms.

In conclusion, we have presented a statistical framework and implementing software for DE analysis of microarray data suggesting that it surpasses conventional workflows in terms of robustness and application range. In addition, many studies are expected to benefit from the cost-saving measure of accurate DE analysis from a small number of replicates (e.g. pilot studies), especially if the pooling of samples is feasible. The accompanying software, available at http://biit.cs.ut.ee/demi/, contains largely automated workflows that will facilitate the analysis of large-scale gene-expression data.

## ACCESSION NUMBERS

Microarray gene expression data generated in this study is available from Gene Expression Omnibus under accession numbers GSE54228 and GSE54229.

## SUPPLEMENTARY DATA

Supplementary Data are available at NAR Online.

## FUNDING

Estonian Science Foundation (9099 to H.L.); Ministry of Science and Education [SF0180125s08 to E.V.]; Estonian Research Council [IUT20-41 to E.V.]; Estonian Research Council [PUT120 to C.A.H.]; Lundbeck Foundation (to C.A.H.); Novo Nordisk Foundation (to C.A.H.); Foundation for Providing Medical Research and the Hartmann Foundation (to C.A.H.); European Social Fund’s Doctoral Studies Internationalization Programme DoRa carried out by Archimedes; Internationalization Programme DoRa carried out by Archimedes Foundation (to S.I. and R.K.). Funding for open access charge: Estonian Research Agency.

*Conflict of interest statement*. None declared.

## Supplementary Material

Supplementary Data
