# Supplementary material for: Estimating differential expression from multiple indicators
Source: Nucleic Acids Res. 2014 Feb 27;42(8):e72. doi: 10.1093/nar/gku158 (PMC4005682; doi:10.1093/nar/gku158)
Supplement: Supplementary Data [file supp_42_8_e72__index.html]

Estimating differential expression from multiple indicators — Estimating differential expression from multiple indicators — Supplementary Data 

# Estimating differential expression from multiple indicators

## Supplementary Data

files

**Files in this Data Supplement:**

- Supplementary Data - zip file
