# Supplementary material for: Estimating differential expression from multiple indicators
Source: Nucleic Acids Res. 2014 Feb 27;42(8):e72. doi: 10.1093/nar/gku158 (PMC4005682; doi:10.1093/nar/gku158)
Supplement: Supplementary Data [file supp_gku158_nar-03204-met-n-2013-File007.zip › Supplementary file 1.docx]

**SUPPLEMENTARY MATERIAL FOR:**

**Estimating differential expression from multiple indicators**

Sten Ilmjärv^1,3^, Christian Ansgar Hundahl^1,4,5^, Riin Reimets^1,4^, Margus Niitsoo^2^, Raivo Kolde^2,3^, Jaak Vilo^2,3^, Eero Vasar^1,4^, Hendrik Luuk^1,4,*^

^1^Institute of Biomedicine and Translational Medicine, University of Tartu, Tartu, Estonia

^2^Department of Computer Science, University of Tartu, Tartu, Estonia

^3^Quretec Ltd, Tartu, Estonia

^4^Centre for Excellence in Translational Medicine, University of Tartu, Tartu, Estonia

^5^Department of Neuroscience and Pharmacology, Faculty of Health Sciences, University of Copenhagen, Copenhagen, Denmark

**Description of supplementary data files**

Supplementary file 2; Format: Excel (.xlsx); Performance evaluation of differential expression estimation pipelines on the MAQC reference samples. The data was used to produce Figure 2A.

Supplementary file 3; Format: Excel (.xlsx); Significance analysis of MCC values obtained by benchmarking differential expression estimation pipelines on the MAQC dataset.

Supplementary file 4; Format: Excel (.xlsx); Effect of hypoxia on gene expression as estimated by DEMI in mouse embryonic fibroblasts exposed to severe hypoxia (N=4).

Supplementary file 5; Format: Excel (.xlsx); Gene ontology annotation enrichment among genes upregulated in mouse embryonic fibroblasts after 24h of severe hypoxia (N=4) as estimated by DEMI.

Supplementary file 6; Format: Excel (.xlsx); Differential expression of genomic regions between prostate cancer cell line LNCaP and normal prostate epithelial cells (N=2) as estimated by DEMI. The data set originates from (1).

Supplementary file 7; Format: Excel (.xlsx); Differential expression analysis identifying monotonic-like temporal gene expression profiles in mildly hypothermic mouse embryonic fibroblasts.

Supplementary file 8; Format: Excel (.xlsx); Differential expression analysis identifying monotonic-like temporal gene expression profiles in normothermic mouse embryonic fibroblasts.

A. Human Genome U133 Plus 2.0 array

|  | Exome | Transcriptome | Genome |
| --- | --- | --- | --- |
| TOTAL HITS | 1251027 | 1752066 | 1767429 |
| + STRAND HITS | 1098827 | 1551269 | 888834 |
| – STRAND HITS | 152200 | 200797 | 878595 |
| PROBES WITH HITS | 431055 | 448726 | 566302 |
| TARGETS WITH HITS | 163865 | 119909 | 23075 |

B. Human Gene 1.0 ST array

|  | Exome | Transcriptome | Genome |
| --- | --- | --- | --- |
| TOTAL HITS | 2732327 | 4114938 | 2925186 |
| + STRAND HITS | 76615 | 85595 | 1440883 |
| – STRAND HITS | 2655712 | 4029343 | 1484303 |
| PROBES WITH HITS | 725178 | 726647 | 783272 |
| TARGETS WITH HITS | 552218 | 178872 | 22894 |

C. Human Exon 1.0 ST array

|  | Exome | Transcriptome | Genome |
| --- | --- | --- | --- |
| TOTAL HITS | 6716529 | 10037710 | 21647909 |
| + STRAND HITS | 1098908 | 1326363 | 10811522 |
| – STRAND HITS | 5617621 | 8711347 | 10836387 |
| PROBES WITH HITS | 2163399 | 2171252 | 5378784 |
| TARGETS WITH HITS | 683699 | 208036 | 23098 |

Table S1. Probe annotation summary based on the number of alignment hits to the Ensembl 73 release of the human exome, transcriptome and genome.

|  |  | N=4 | | N=3 | | N=2 | |
| --- | --- | --- | --- | --- | --- | --- | --- |
| Normalization | DE | mean | sem | mean | sem | mean | sem |
| relative ranking | DEMI | 1.6E-14 | NA | 7.0E-14 | 3.0E-14 | 1.2E-12 | 5.6E-13 |
| DFW | Limma | 5.3E-16 | NA | 7.2E-13 | 7.0E-13 | 0.619 | 0.081 |
| DFW | Rankprod | 1.7E-18 | NA | 2.3E-13 | 1.4E-13 | 4.8E-07 | 2.7E-07 |
| FARMS | Limma | 1.9E-13 | NA | 2.1E-12 | 2.0E-12 | 0.451 | 0.083 |
| FARMS | Rankprod | 4.2E-12 | NA | 4.3E-08 | 3.8E-08 | 0.003 | 0.001 |
| PLIER | Limma | 4.5E-12 | NA | 0.9 | 0.1 | 1 | 0 |
| PLIER | Rankprod | 3.0E-01 | NA | 1 | 0 | 1 | 0 |
| RMA | Limma | 5.6E-11 | NA | 1.0E-11 | 8.7E-12 | 0.478 | 0.083 |
| RMA | Rankprod | 1.6E-11 | NA | 1.9E-10 | 7.0E-11 | 1.3E-04 | 9.4E-05 |

Table S2. Enrichment of mouse orthologs of HIF-2 targets among significantly up-regulated genes in mouse embryonic fibroblasts exposed to 1% O_2_ for 24h. Differential gene expression was estimated by nine pipelines composed of various normalization and differential expression estimation methods. The data is presented as mean and standard error of hypergeometric p-values from all possible comparisons between subsets of size N of the hypoxic and normoxic groups (original N=4). Abbreviations: differential expression (DE), sample size (N), standard error of mean (sem), not available (NA)

**Supplementary references**

1. Coolen,M.W., Stirzaker,C., Song,J.Z., Statham,A.L., Kassir,Z., Moreno,C.S., Young,A.N., Varma,V., Speed,T.P., Cowley,M., et al. (2010) Consolidation of the cancer genome into domains of repressive chromatin by long-range epigenetic silencing (LRES) reduces transcriptional plasticity. *Nat. Cell Biol.*, **12**, 235–246.
